# A new 37- level inverter with reduced switches for renewable energy applications

**DOI:** 10.1038/s41598-025-31963-6

**Published:** 2025-12-13

**Authors:** Shubhi Shukla, Vidushi Goel, C. Dhanamjayulu

**Affiliations:** School of Electrical Engineering, Vellore Institute of Technology, Vellore, India

**Keywords:** Total harmonic distortion (THD), Total standing voltage (TSV), Cost function (CF).Multilevel inverter (MLI), Energy science and technology, Engineering

## Abstract

Multilevel inverters (MLIs) are now crucial in producing high-quality output waveforms due to their modularity and efficiency. This paper presents a novel 37- level MLI topology with a reduced number of switches and sources. The proposed design offers several advantages, including lower total harmonic distortion (THD) of 1.21% in hardware and 0.8% in simulation, high efficiency of 93.26%, reduced total standing voltage of 18 V_DC_), and an improved component-per-level ratio compared to existing MLIs. The inverter performance is validated using MATLAB/Simulink and a laboratory prototype controlled by a dSPACE system under different load conditions. Results confirm that the proposed inverter maintains stable operation during dynamic load changes and provides a cost-effective, compact, and reliable solution for renewable energy and electric vehicle applications.

## Introduction

The multilevel inverter (MLI) topologies such as CHB, NPC, and FC have been developed still face challenges like increased switch count, complex control, and higher total standing voltage (TSV) at higher levels. These issues lead to reduced efficiency, reliability, and cost-effectiveness. To overcome these limitations, the proposed asymmetric 37-level inverter achieves more voltage levels using fewer switches and reduced TSV, thereby enhancing efficiency and lowering THD for renewable and electric vehicle applications^1^. The applications of PV systems in a plethora of applications such as grid electricity generation, electric vehicles (EVs), and power distribution in industry have generated the demand for efficient power conversion technology^2–4^. One of the most vital parts of the process is the inverter that enables the power conversion of the DC electricity generated by solar PV panels into AC electricity compatible with contemporary electrical loads and aircraft applications^5,6^. Nevertheless, for high-power applications, traditional voltage source inverters (VSIs) efficiency and reliability are hampered by serious drawbacks such as high switching losses, high total harmonic distortion (THD)^7,8^, and high electromagnetic interference (EMI)^9^. Multilevel inverters (MLIs) have been recognized to be the better alternative to these challenges with higher power quality, lower switching stress, and higher efficiency; therefore, they are a choice for power conversion in industrial and renewable energy applications^10–15^.

Baker and Bannister initially developed multilevel inverters in the 1970 s to enhance power conversion efficiency using multiple DC sources and semiconductor switches to generate stepped voltage waveforms^16^. Numerous different MLI topologies have been suggested since then, including the cascaded H-Bridge (CHB)^17–19^, neutral-point clamped (NPC)^20–22^, and flying capacitor (FC) configurations^23–25^. Due to its modularity, scalability, and minimal dependence on high-power semiconductor switches, the CHB-MLI has been the most widely used among them^26^. CHB-MLIs are highly efficient for high-power applications since they do not need extra voltage-balancing components, unlike NPC-MLIs, which need clamping diodes to provide voltage balance between series-connected capacitors^10–28^. While NPC-MLIs have improved voltage sharing, their high voltage-level complexity makes them susceptible to operational disadvantages such as non-uniform voltage stress distribution among power devices^29^. FC-MLIs with floating capacitors rather than diodes to balance voltage also need a lot of capacitors, making them unsuitable for high-voltage applications. The largest drawback of traditional MLIs, despite their benefits, is that they do not handle the growing number of components as output voltage levels increase. Higher-level MLIs require more isolated DC sources, gate driver circuits, and semiconductor switches, which increases system cost, switching losses, and control approach complexity^30–32^.

To overcome these limitations, researchers have investigated asymmetric MLI topologies that employ non-equal DC voltage sources to obtain maximum voltage levels with fewer switches and passive components^33^. This design method increases cost-effectiveness, efficiency, and system compactness, and therefore asymmetric MLIs are ideal for power applications in the present time^34^. Asymmetric MLIs can generate the same or even greater voltage levels with fewer components than symmetric MLIs, which employ equal DC voltage sources and additional switches to generate different voltages. According to research, asymmetric configurations greatly reduce conduction losses and total standing voltage (TSV), increasing MLIs’ power efficiency and harmonic reduction capability^35^. more MLI structures emphasizing lower semiconductor stress, lower cost, and higher operating reliability are the outcome of the increased demand for power electronic systems to be optimized^36–41^.

To meet these changing needs, this paper introduces a new 37-level asymmetrical MLI with improved efficiency, reduced total harmonic distortion, reduced semiconductor switches, and reduced total standing voltage. The new topology utilizes five asymmetrical DC sources, four bidirectional switches, and eight unidirectional switches, which together provide a high-efficiency, low-cost solution with low THD. In comparison with traditional MLI topologies, the proposed 37-level inverter has some benefits, such as enhanced power quality, reduced semiconductor usage, and enhanced efficiency, which makes it a suitable candidate for high-power applications. The proposed topology minimizes TSV by utilizing an optimal switch and source configuration, which minimizes the stress on semiconductor devices and enhances system reliability. Moreover, the stepped output waveform provides less harmonic distortion, which eliminates the need for complex filtering devices and enhances the overall efficiency of the inverter. An optimized control scheme also allows the inverter to provide a constant voltage output even when loads change, which makes it more appealing for electric car power systems, industrial motor drives, and renewable energy integration^42–44^.

The new 37-level inverter is contrasted with traditional MLI topologies on key parameters like THD, TSV, efficiency, and power loss for comparison of its performance. Simulation is carried out to analyze its efficiency, and the design is also experimented to check its viability for applications. Findings show the superiority of the new MLI as a future device for high power conversion efficiency with reduced harmonic distortion. Breaking the limitations of traditional MLIs, this research provides a new boost to the development of power electronics and its applications to electric vehicle charging, renewable energy integration, active power filtering, and uninterruptible power supplies. The 37- output levels were obtained with four bidirectional & eight unidirectional switches, as discussed^45^. The bidirectional switch $\:{S}_{c}$ can be replaced with a unidirectional switch to generate the same output levels.

Recent study has investigated reduced-component and switched-capacitor-based MLI to improve efficiency and reduce device count. Debela et al.^46^ proposed a grid-connected boost multilevel inverter (BMLI) with a simplified configuration, achieving enhanced performance in renewable energy applications. Furthermore^47^, Evaluated an H-bridge-less grid-tied inverter emphasizing minimized TSV. These works support the growing trend toward optimized MLIs, which motivates the development of the proposed 37-level inverter with further reductions in TSV, THD, and switch count. These advancements reinforce the need for novel inverter designs with reduced switch count and improved harmonic performance, which motivates the proposed 37-level inverter presented in this work.

 In the realm of solar PV energy systems, these advancements are pivotal, enhancing the capacity and efficiency of Electric Vehicles (EVs) and FACTS. The proposed 37 level MLI architecture, detailed in this paper, is accompanied by comprehensive circuit schematics and switching tables in Sect. 2. Sect. 3 presents a comparison of simulated and measured results from hardware implementations. Sect. 4 evaluates parameters such as TSV, cost-efficiency, power losses, and overall system efficiency. Finally, Sect. 5 discusses the implications of these findings and outlines avenues for future research.

The novelty of the proposed work lies in the development of an asymmetric 37-level multilevel inverter (MLI) topology that achieves a significant reduction in switch count, total standing voltage (TSV), and harmonic distortion, while maintaining high efficiency. Compared to existing topologies, the design uniquely balances performance and economic feasibility with only 12 switches (including 4 bidirectional), 5 unequal DC sources, and reduced component-per-level ratio. The inverter’s performance is validated through both simulation and hardware using dSPACE RTI 1104, highlighting its practical applicability for renewable energy and EV systems.

## Proposed asymmetrical 37-Level MLI topology

**Fig. 1:**
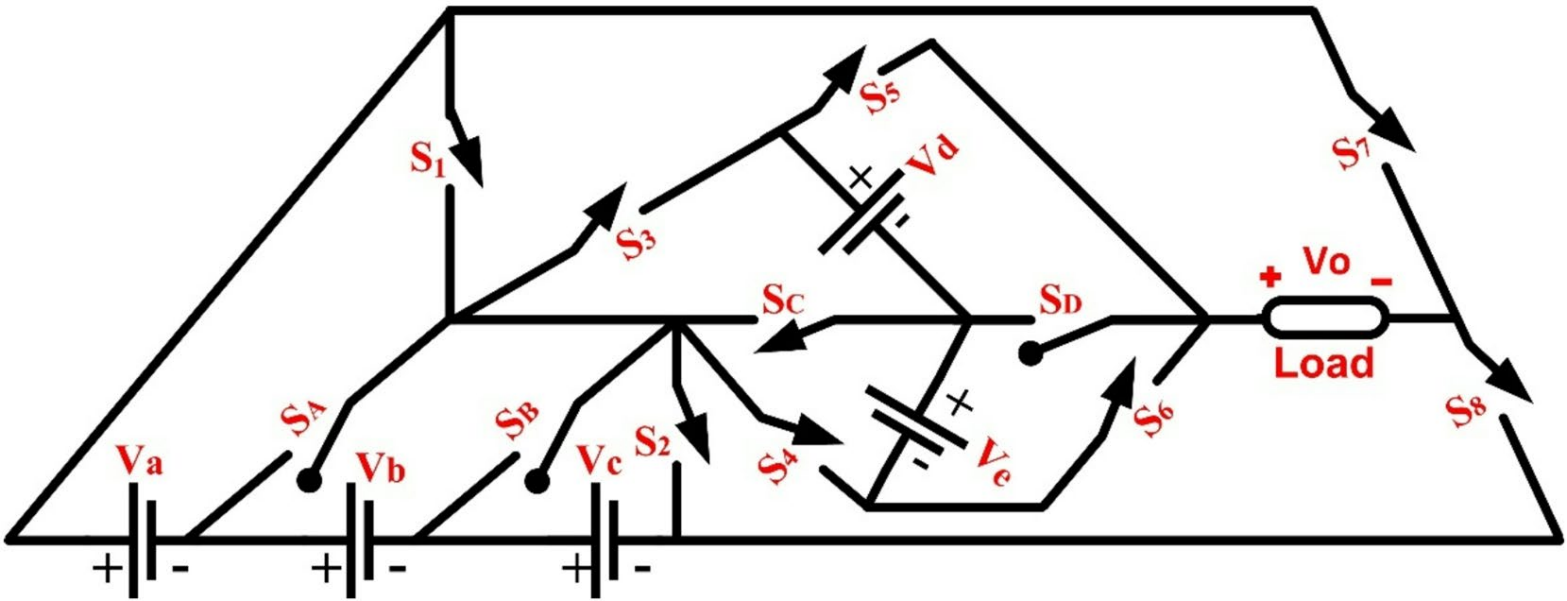
Novel 37- level MLI.

The proposed MLI with reduced components is presented in Fig. 1, which features three bidirectional and eight unidirectional switches to achieve the maximum possible output levels.

The selection of components as follows:

The general cells (*m*) & supply sources (*n*) are required to estimate the parameters of the proposed architecture.

The sources $\:{(R}_{DC})$ can be evaluated with unequal sources combination

1 $$\:{\:R}_{DC}={1+2}^{m}$$

$\:{R}_{DC}$ is obtained with Eq. (1)

$$\:{R}_{DC}={1+2}^{2}=1+4=5$$

The number of voltage levels (V_*PL*_) is calculated

2 $$\:{V}_{PL}=1+{6}^{m}$$

$\:{V}_{PL}$ can be obtained with Eq. (2)

$$\:{V}_{PL}={1+6}^{2}=1+36=37$$

The required switches (*R*_*SW*_) can be estimated

3 $$\:{R}_{SW}={4}^{m}-4$$

$\:{R}_{Sw}$ is designed with Eq. (3)

$$\:{R}_{Sw}={4}^{2}-4=12$$

The magnitude is designed

4 $$\:{V}_{po}=\frac{\left[{6}^{m}+1\right]+1}{2}\mathrm{*}Rdc$$

The voltage is estimated with Eq. (4)

$$\:{V}_{po}=\frac{\left[{6}^{2}+1\right]+1}{2}\mathrm{*}22.5=405\:V$$

Where *m = 2 and* source voltage are $\:22.5V$.

**Fig. 2:**
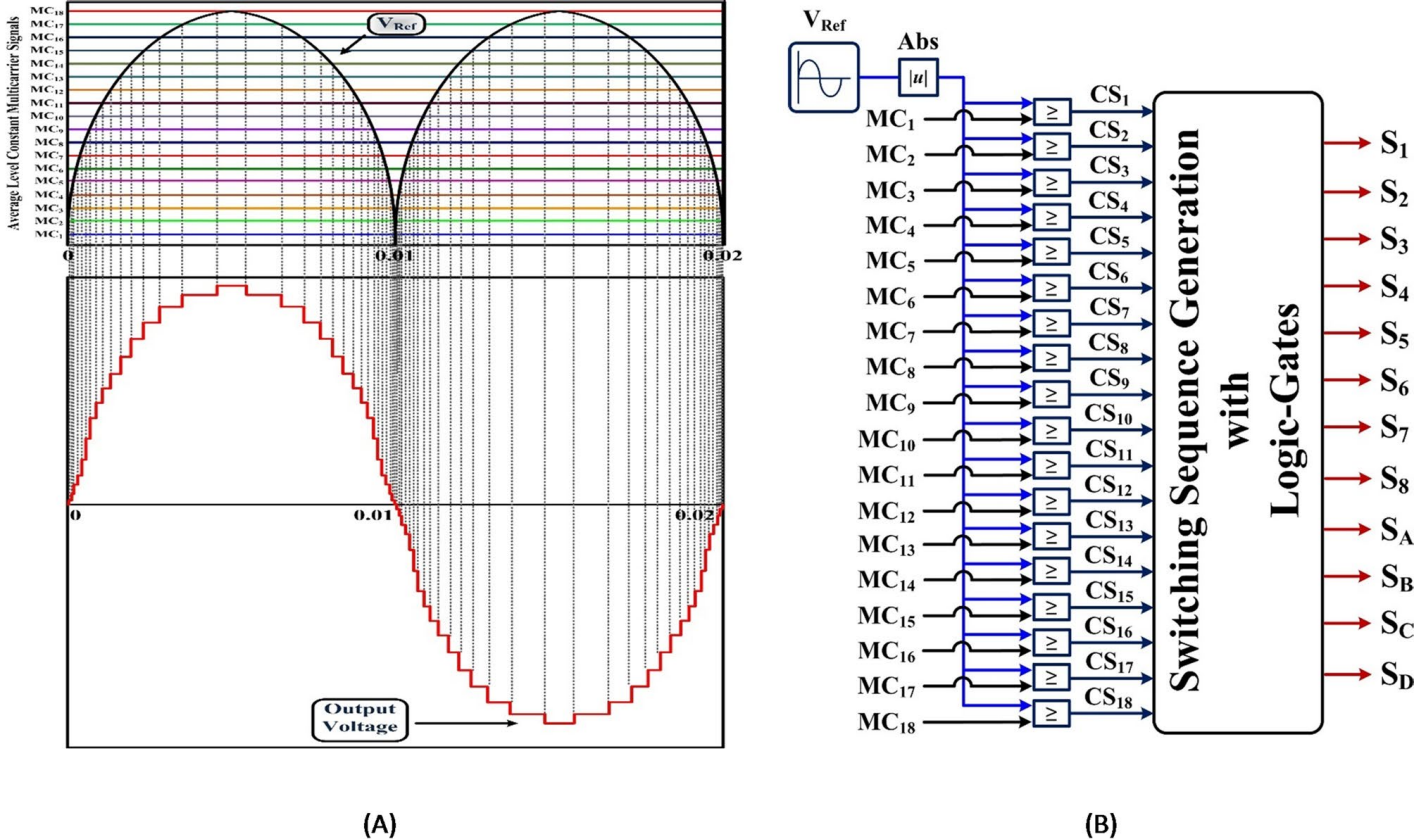
(a). Expected stepped voltage output waveform (b). Switching Sequence generation.

Using the equations mentioned above, the estimated components are designed for the proposed MLI. It can be extended to increase the various voltage levels with different voltage source combinations. Figure 1 shows a newly developed 37- level with reduced circuit power sources and switches for the generation of required high-quality output waveforms. The equal power sources such as $\:{V}_{a}\to\:{V}_{b}\to\:{V}_{c}\to\:22.5V={R}_{dc}$, $\:{V}_{d}\to\:157.5=7{R}_{dc}$and $\:{V}_{e}\to\:180V=8{R}_{dc}$with values of resistance and inductor $\:100\:\varOmega\:,\:150\:mH,\:and\:200\:mH$ respectively are implemented. The switches $\:{S}_{D}\to\:{S}_{B}\to\:{S}_{A}$ allow the current path for both the directions and $\:{S}_{8}\to\:{\to\:S}_{7}\to\:{\to\:S}_{6}\to\:\to\:{S}_{5}\to\:\to\:{S}_{4}\to\:{\to\:S}_{3}\to\:\to\:{S}_{2}\to\:\to\:{S}_{1}$ $\:\to\:\to\:{S}_{c}$will conduct in only one direction. The required conduction of states is given in Table. I and its expected stepped voltage output waveform is shown in Fig. 2(a).

Although the switching sequence involves multiple states, the overall circuit offers clear benefits in terms of reduced TSV, lower switch count per level, and improved efficiency. The control strategy can be readily implemented on digital platforms such as dSPACE, making the added switching complexity acceptable for practical applications.

The proposed topology’s control strategy is implemented using a level-shifted Pulse Width Modulation (PWM) technique. A dSPACE RTI 1104 controller is used to generate PWM signals based on real-time load sensing. The modulation strategy ensures proper switching of unidirectional and bidirectional switches to synthesize the desired 37-level output. Figure 2(b) shows the switching Sequence generation of the modulation control implemented. The logic gate-based system that uses timing signals from the comparator to generate pulses in accordance with switch operation criteria. These logics are essential for transforming time signals into a logic pattern that the switch operates to produce.

The Table 1 is a switching table which guides the selection of switches for each voltage level.

**Table 1:** Proposed 37- MLI switch conduction.

L	Switch conduction directions											
	^‘^S_A_^’^	^‘^S_B_^’^	^‘^S_C_^’^	^‘^S_D_^’^	^‘^S_1_^’^	^‘^S_2_^’^	^‘^S_3_^’^	^‘^S_4_^’^	^‘^S_5_^’^	^‘^S_6_^’^	^‘^S_7_^’^	^‘^S_8_^’^
L1					^**‘**^↓			^**‘**^↓	^**‘**^→			^**‘**^↓
L2	^**‘**^→							^**‘**^↓	^**‘**^→			^**‘**^↓
L3		^**‘**^→						^**‘**^↓	^**‘**^→			^**‘**^↓
L4						‘↓		^**‘**^↓	^**‘**^→			^**‘**^↓
L5	^**‘**^→							^**‘**^↓	^**‘**^→			^**‘**^↓
L6		‘→						^**‘**^↓	^**‘**^→		^**‘**^↓	
L7						‘↓		^**‘**^↓	^**‘**^→		^**‘**^↓	
L8				←^**‘**^	^**‘**^↓			^**‘**^↓				^**‘**^↓
L9			‘→		‘↓				^**‘**^→			^**‘**^↓
L10	^**‘**^→		^**‘**^→						^**‘**^→			^**‘**^↓
L11		^**‘**^→	^**‘**^→						^**‘**^→			^**‘**^↓
L12			^**‘**^→			^**‘**^↓			^**‘**^→			^**‘**^↓
L13	^**‘**^→		^**‘**^→						^**‘**^→		^**‘**^↓	
L14		^**‘**^→	^**‘**^→						^**‘**^→		^**‘**^↓	
L15			^**‘**^→			^**‘**^↓			^**‘**^→		^**‘**^↓	
L16					^**‘**^↓		^**‘**^→		^**‘**^→			^**‘**^↓
L17	^**‘**^→						^**‘**^→		^**‘**^→			^**‘**^↓
L18		^**‘**^→						↓		^**‘**^→		^**‘**^↓
L19						^**‘**^↓		↓		^**‘**^→		^**‘**^↓
L20	^**‘**^→						‘→		‘→		^**‘**^↓	
L21		^**‘**^→						^**‘**^↓		^**‘**^→	^**‘**^↓	
L22						^**‘**^↓		^**‘**^↓		‘→	^**‘**^↓	
L23				←’	↓^**‘**^		^**‘**^→					^**‘**^↓
L24	^**‘**^→			←’			^**‘**^→					^**‘**^↓
L25		^**‘**^→		←’			^**‘**^→					^**‘**^↓
L26				←’	^**‘**^↓		‘→				^**‘**^↓	
L27	^**‘**^→			←’			‘→				^**‘**^↓	
L28	→		^**‘**^→							‘→	‘↓	
L29		^**‘**^→	^**‘**^→							‘→	‘↓	
L30			^**‘**^→			↓				‘→	^**‘**^↓	
L31					^**‘**^↓		^**‘**^→			‘→		^**‘**^↓
L32	^**‘**^→						‘→			‘→		‘↓
L33		^**‘**^→					‘→			‘→		‘↓
L34					^**‘**^↓		‘→			‘→	‘↓	
L35	^**‘**^→						‘→			‘→	‘↓	
L36		^**‘**^→					‘→			‘→	‘↓	
L37						^**‘**^↓	^**‘**^→			‘→	‘↓	

^**‘**^→ indicates the direction of the conducting switch & ‘L’ is levels.

The maximum peak voltage will be generated with all active sources are.

**Fig. 3:**
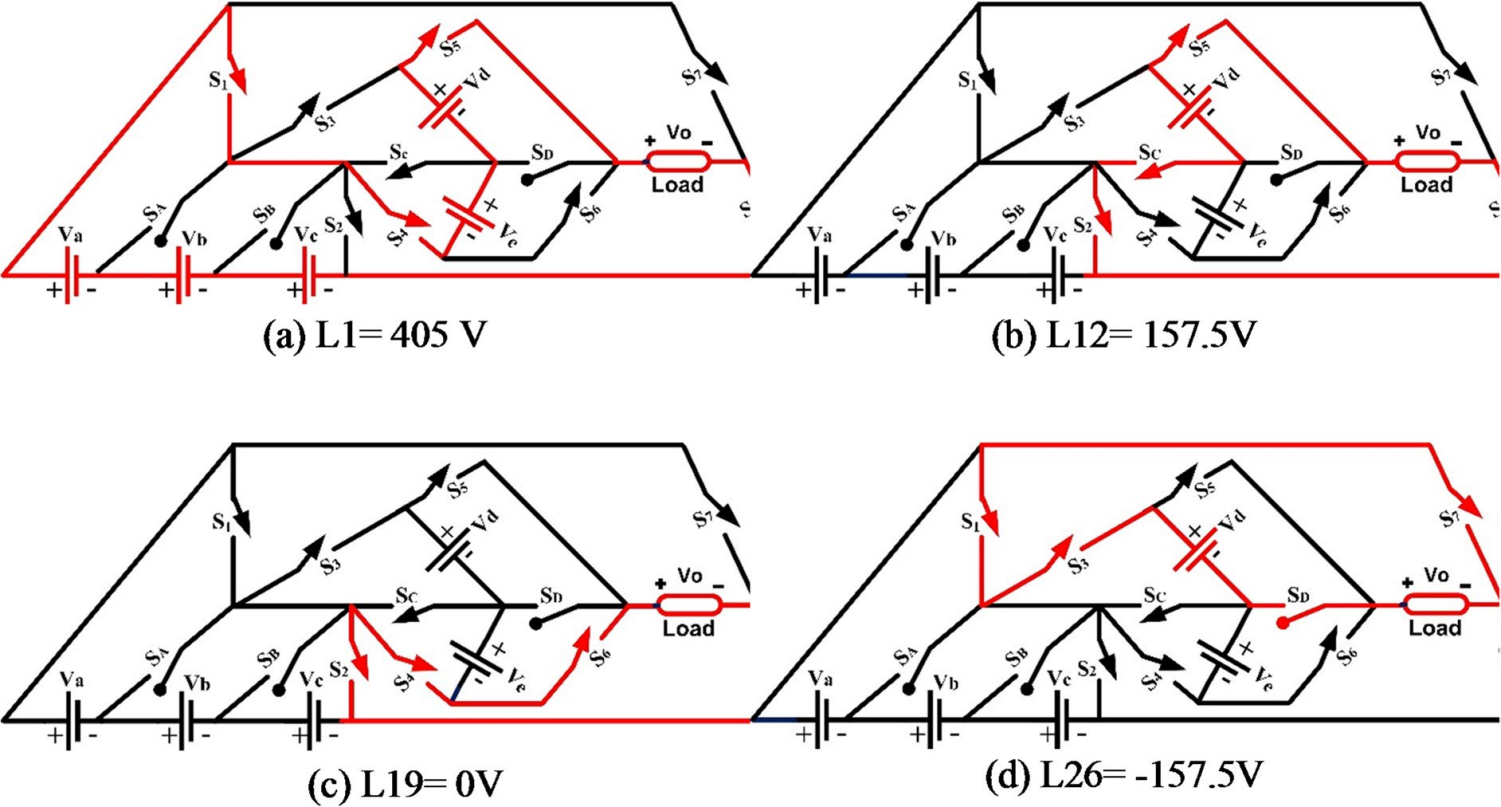
37- MLI current paths.

In state-1: 405 V, + 18V_DC_, the current flows as follows: V_a_-S_1_-S_4_-V_e_-V_d_-S_5_-L-S_8_-V_c_-V_b_-V_a_, as shown in Fig. 3. All DC sources are utilized to power the circuit (V_a_ +V_b_ + V_c_ + V_d_ + V_e_). For the purpose of creating extra positive voltages and zero voltage levels, the load current Io flows via a sequence of switches S_1_, S_4_, S_5_, and S_8_. Similar to state-19: 0 V, 0V_DC_, none of the sources (V_a_, V_b_, V_c_, V_d_, and V_e_) are supplying DC power to the system. Thus, L-S_8_ - S_2_ - S_4_ - S_6_ - L is the wiring path for the load current Io. A negative voltage of −2V_DC_ is generated when all seven of the S_B_, S_4_, S_6_, and S_7_ are active in state-21: −45 V operation, and the load current Io follows the following conductor path: L-S_6_ -S_4_ - S_B_ - V_b_ - V_a_ - S_7_ - L.

## 3. Results analysis

**Fig. 4:**
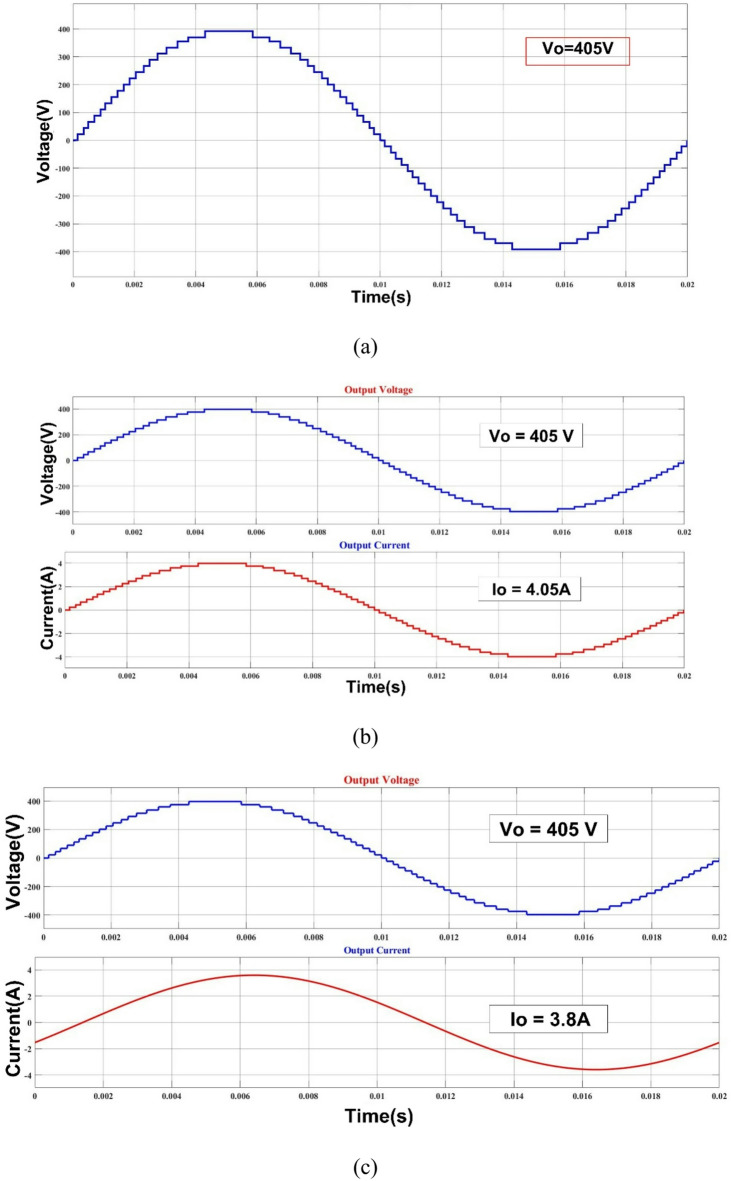

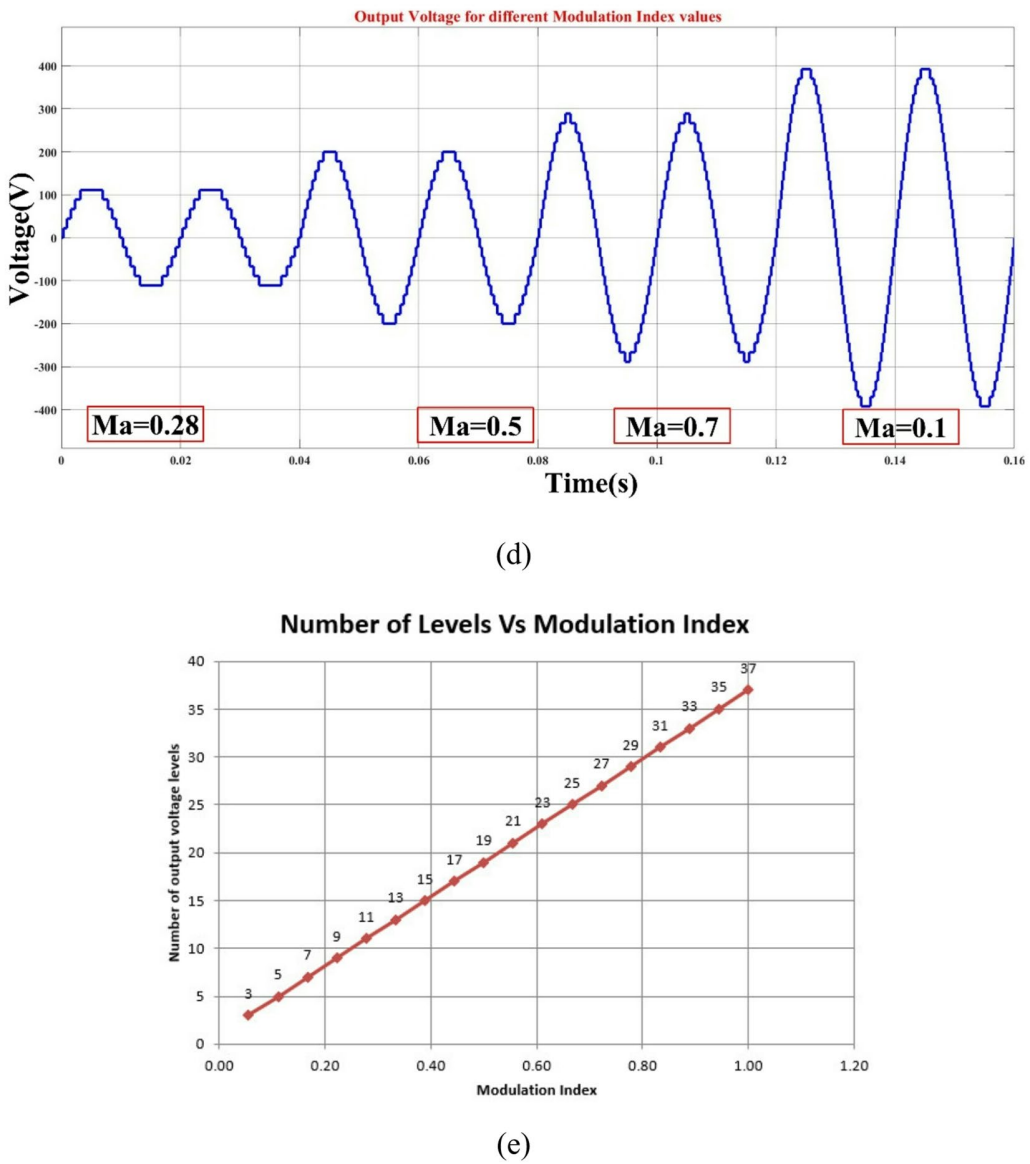
37- level MLI. (a) Output voltage (Vo), (b) R Load (Vo & Io), (c) L Load (Vo & Io), (d) Simulation output voltage of Modulation Index vs. Output Voltage and (e) Modulation Index vs. No. of Levels.

**Fig. 5:**
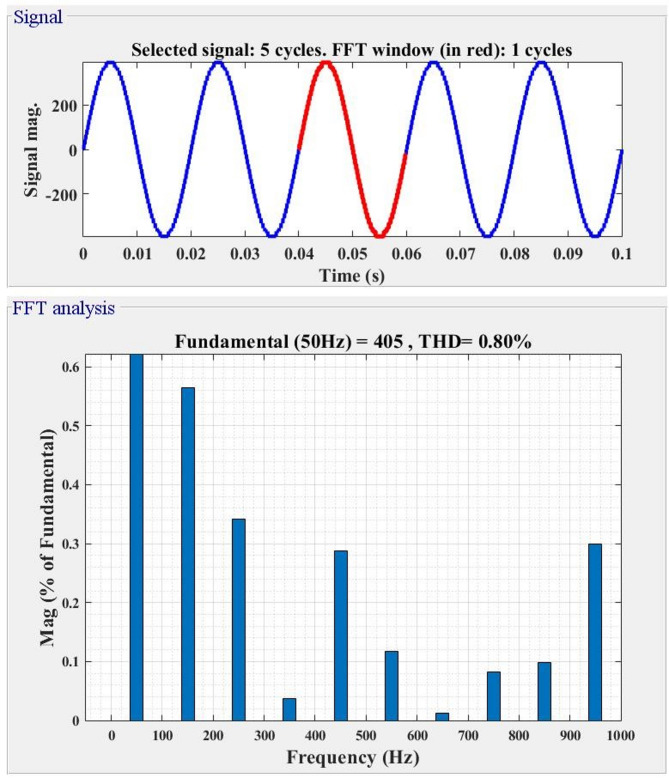
Simulation THD of MLI.

V_DC_=V_a_=V_b_=V_c_=22.5 V, V_d_=157.5 V, and V_e_=180 V are the input DC sources that are used to test the architecture for the proposed MLI that is being used. The waveforms of the voltage and current outputs from MATLAB Simulink are plotted in Fig. 4(a-d) in the appropriate order. As shown in Fig. 4(a), the peak voltage is 405 V, the load current is 4.05 A, and the resistance of the load is shown to be 100 Ω. For the resistive load of 100 Ω and the inductive load of 150 mH, the output voltage (Vo) and current (Io) are shown in Fig. 4(b) and (c), respectively. In Fig. 4(d) and Fig. 4(e), the output voltage levels are depicted for a variety of modulation index (Ma) values. Furthermore, the THD yield is 0.80%, as can be seen in Fig. 5. The load, switching frequency, reference frequency, and DC voltage sources that are recommended for the architecture are all presented in Table 2.

**Table 2:** Specifications of the components.

Components/Parameters	Ratings/Type
Driver Circuit	TLP250
R-load	100 Ω
Switching frequency	2000 Hz
DC sources	500 V/Programmable
IGBTs	600 V,75 A/(CM75DU-12 H)
dSPACE Controller	RTI 1104
Reference frequency	50 Hz

**Fig. 6:**
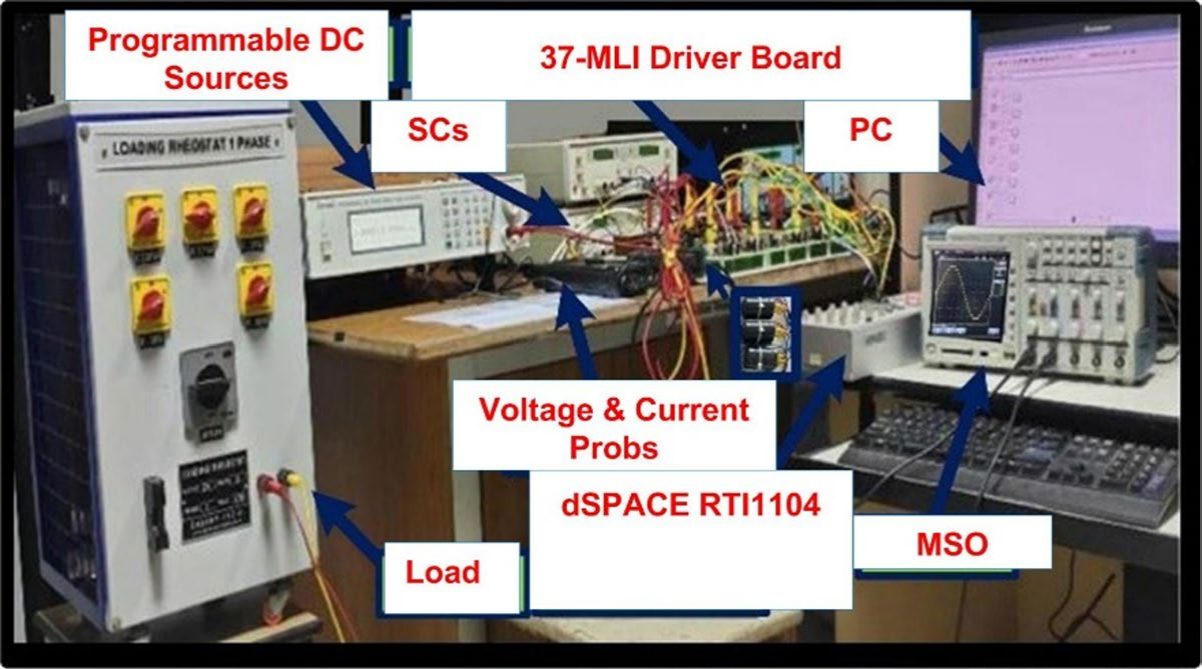
Laboratory Set-up.

**Fig. 7:**
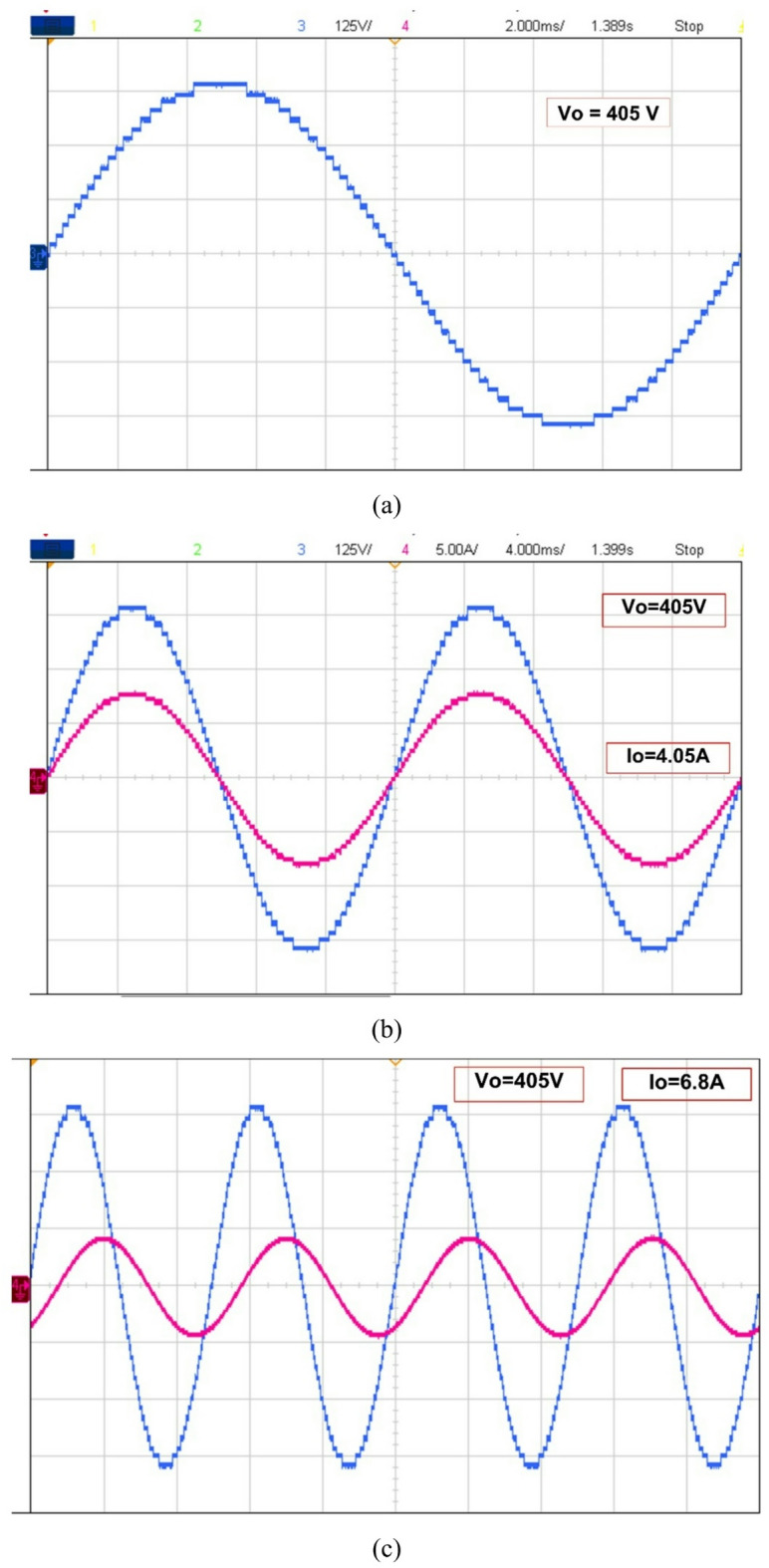

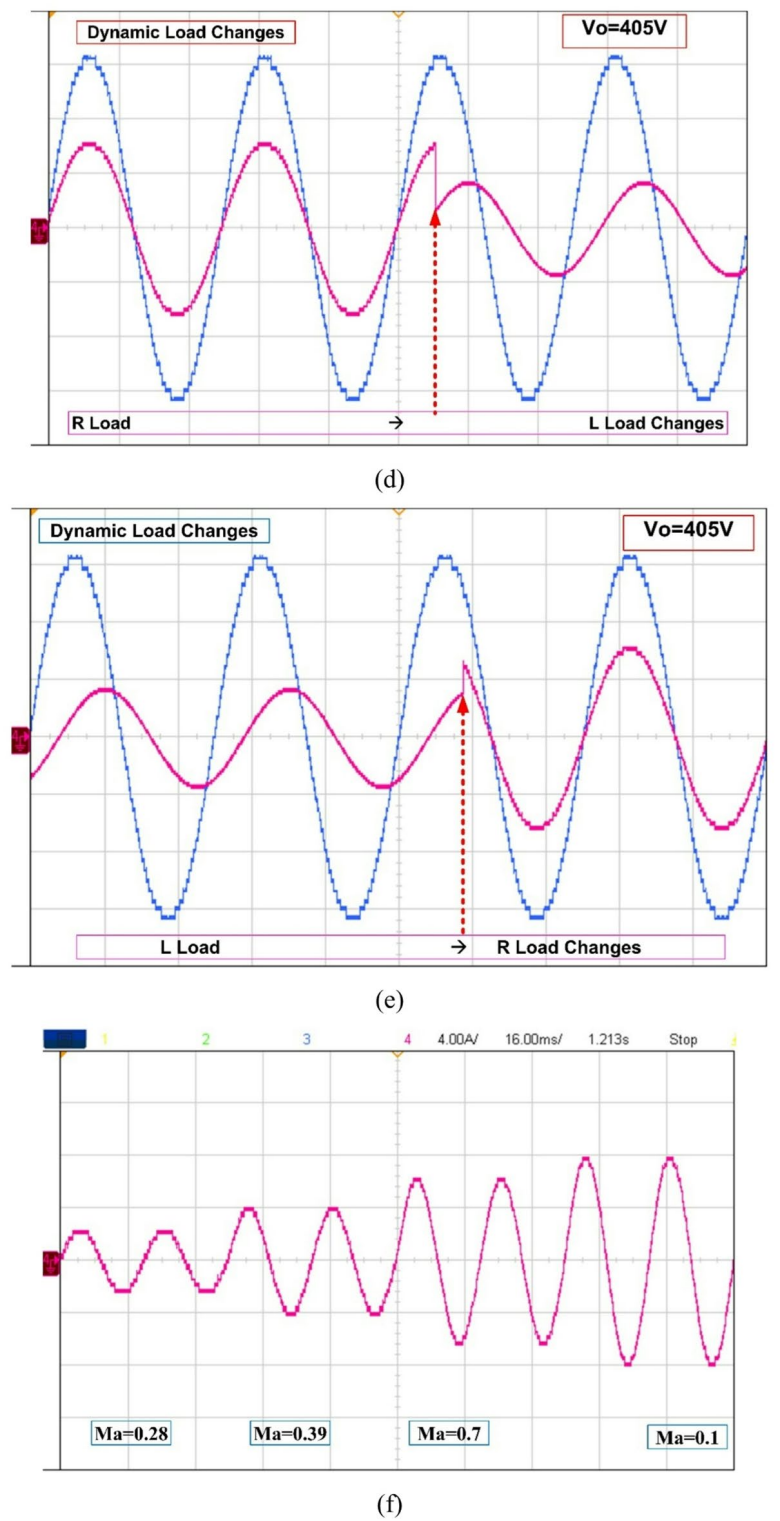
Hardware results of 37- level MLI, (a) output voltage waveform. (b) R load results (c) Motor load results, (d) Dynamic RL load changes, (e) Dynamic LR load changes, and (f) Levels Vs MI.

A working model of an experimental setup for a 37- level MLI configuration has been created and validated. Figure 6 shows the experimental setup for the 37- level inverter architecture that has been presented. An experimental kit prototype is utilized to implement the required configuration using dSPACE RTI 1104. The THD value of 0.80% corresponds to simulation under ideal conditions; the hardware prototype (Fig. 7) yields a measured THD of 1.21%, which validates the proposed design under practical operation.

The experimental setup includes opto-isolated gate driver circuits (TLP250) with dead-time insertion to prevent shoot-through. Heat sinks and thermal paste were applied to high-power IGBTs for thermal protection. The system design also considers scalability, where the modular DC source configuration allows extension to higher voltage levels without modifying the control structure.

**Fig. 8:**
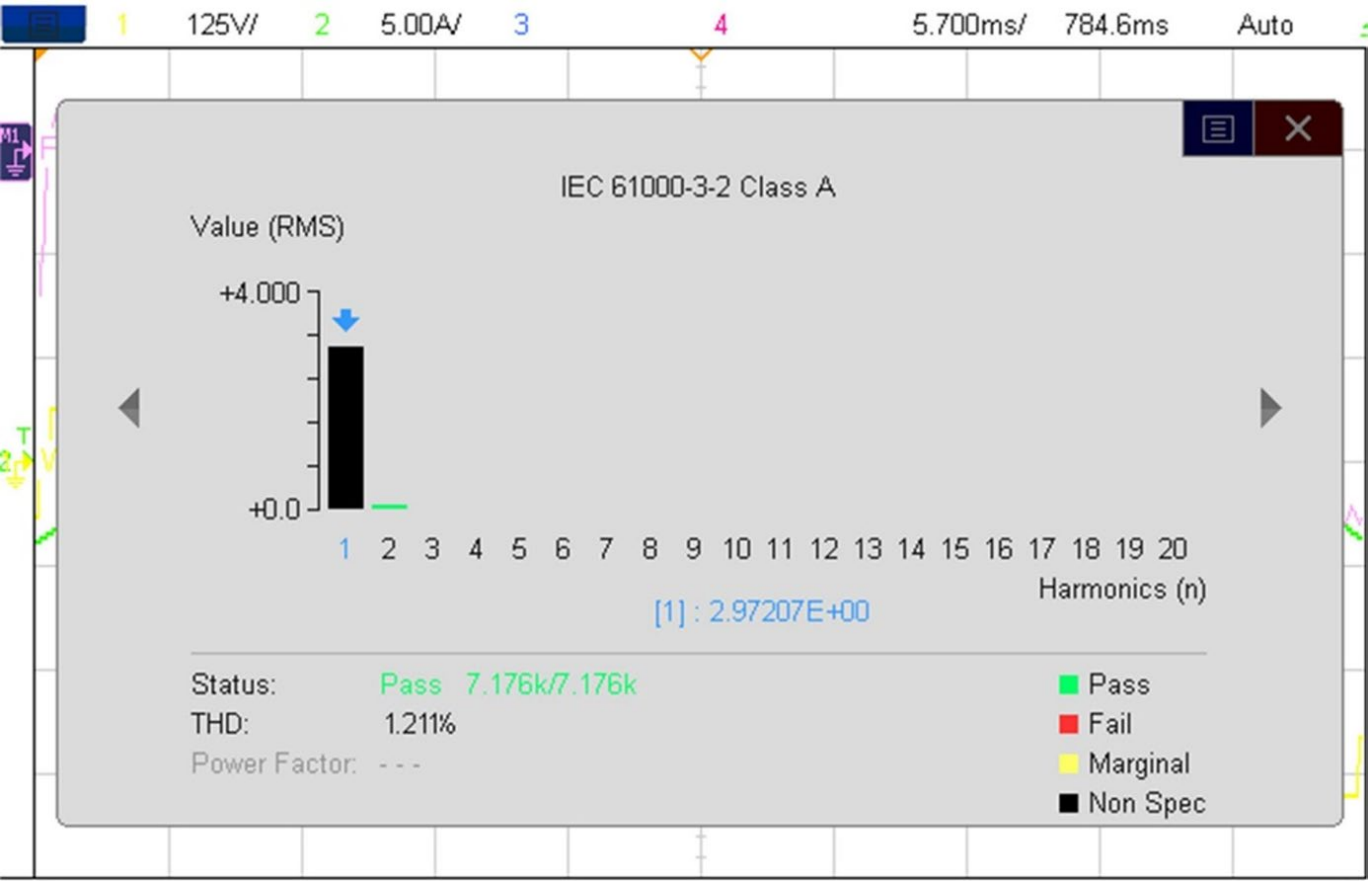
Experimental THD of the suggested MLI.

The recommended configuration was tested in MATLAB/Simulink before being sent to the dSPACE controller via digital I/O ports. It worked well thereafter. The gate driver enhances the PWM circuit’s design and can operate on voltages ranging from 4 to 16 V. The power semiconductor switches are activated utilizing a 15-volt pulse. Figure 8 displays the hardware’s results from the resistive load testing. The Fig. 8(a) & 7(b) is representing the prototype results of R load with 405 V output voltage and load current I_0_ = 4.05 A. The Fig. 8(c), (d) &€ shows the output current lags behind the output voltage of inverter (V_0_ = 405 V & I_0_ = 6.8 A) and output levels vs. modulation index is presented in Fig. 8(f). The voltage THD is 1.21%, as displayed in Fig. 7.

**Table 3:** Proposed inverter experimental and simulation results comparisons.

Parameter	Simulation Outcome	Hardware Outcome
Output Voltage (V_o)_	405 V	405 V
Output Current (I_o)_	4.05 A	4.05 A
THD	0.80%	1.21%

`The measured voltage THD is 1.21%, as indicated in Table 3. In accordance with IEEE standards, the suggested configuration can produce a scalable voltage level with minimal components and THD.

## Estimation and comparison of the proposed MLI performance parameters

### Estimation of total power losses (P_total loss_) and efficiency (η%)

Conduction losses (*P*_*CDi*_) and switching losses (*P*_*SW*_) are two examples of the power losses that inverters mostly have to deal with. Taking into account the losses in the current conduction state of the switch IGBT (*P*_*IGBT*_) and the anti-parallel diode (*P*_*ADi*_) yields net amount of conduction losses, which are represented as

5 $$\:{{P}_{CDi}\left(\mathrm{t}\right)=\mathrm{P}}_{\mathrm{I}\mathrm{G}\mathrm{B}\mathrm{T}}\left(t\right)+{P}_{ADi}\left(t\right)$$

6 $$\:{{P}_{CDi}\left(\mathrm{t}\right)=\left\{\right[\mathrm{V}}_{\mathrm{I}\mathrm{G}\mathrm{B}\mathrm{T}}+{R}_{DI}{i}_{n}^{\beta\:}\left(t\right)]+[{V}_{DI}+{R}_{DI}{i}_{n}\left(t\right)\left]\right\}{i}_{n}\left(t\right)$$

The threshold voltages of switch (*V*_*IGBT*_), current (*i*_*n*_), and on/off switch (in) are represented below. Given that the switches (*N*_*IGBT*_) & diodes (*N*_*Di*_) are activated at the same time intervals (t), where *R*_*IGBT*_ and *R*_*DI*_ denote the on-state resistance of the IGBT and diode, respectively, and$\:\:\beta\:\:$stands for the switch constant. The power loss on average as follows

7 $$\:{{P}_{CDi}=\frac{1}{2\pi\:}{\int\:}_{0}^{2\pi\:}\left\{{N}_{IGBT\:}\right(t)\mathrm{P}}_{\mathrm{I}\mathrm{G}\mathrm{B}\mathrm{T}}\left(t\right)+{N}_{Di}\left(t\right){P}_{ADi}\left(t\right)\}dt$$

The energy losses that occur during power consumption include energy turn-on (E_on_) and turn-off (E_off_) for IGBT turn-on and off states.

8 $$\:{E}_{off-time}=\frac{1}{6}\:{V}_{IGBTj}I{t}_{off}$$

9 $$\:{E}_{on-time}=\frac{1}{6}\:{V}_{IGBTj}{I}^{{\prime\:}}{t}_{on}$$

The loss in switch is *j*, the *t*_*on*_ *& t*_*off*_ are the turn–on/off, *E*_*off−time*_ and *E*_*on−time*,,_I *& I’* of the switches respectively.

10 $$\:{P}_{SW}=f\left\{\sum\:_{j=1}^{{N}_{IGBT}}{\left[\sum\:_{j=1}^{{N}_{on-time\:j}}{E}_{on-time\:j}+\sum\:_{j=1}^{{N}_{off-time\:j}}{E}_{off-time\:j}\right]}_{\:}\right\}$$

The *N*_*on−time j*_ and *N*_*off−time j*_ are the switch turn-on/off j^th^ time intervals with fundamental (*f*) with complete cycle.

The inverter’s complete Power losses (*P*_*total losses*_) is estimated^47^

11 $$\:{{P}_{Total\:losses}={P}_{CDi}+P}_{SW}$$

12 $$\:{P}_{outp}={V}_{rms}\mathrm{*}{I}_{rms}$$

The inverter’s overall efficiency (η%) is obtained

13 $$\:\eta\:\left(\mathrm{\%}\right)=\frac{{P}_{outp}}{{P}_{inpp}}=\frac{{P}_{outp}}{{P}_{outp}+{P}_{Total\:losses}}$$

The inverter’s output and input powers are denoted by *P*_*outn*_ and *P*_*inpp*_, respectively. Table 4 has a detailed discussion of the power losses and efficiency values that were determined.

**Table 4:** List of power loss calculations of the proposed MLI.

Voltage (V_rms_)	286.42 V
R-load	100 Ω
Current (I_rms_)	2.86 A
Switching losses (*P*_*SW*_*)*	0.046 W
Conduction losses *(P*_*CDi*_*)*	4.89 W
Total losses (*P*_*Total losses*_*)*	59.23 W
Output power (*P*_*outn*_*)*	819.16 W
Input power (*P*_*inpp*_*)*	878.36 W
Efficiency (% η)	93.26

### Total standing voltage (TSV) Estimation

A representation of the voltage that is being blocked is the ‘pressure’ that is across the switch. “Unidirectional” and “bidirectional” switches have different “voltage strains,” which means that they are not the same. The maximum output voltage is calibrated to 405 V when the highest performance level (V_0 MAX_) is used. The presented inverter, the equivalent voltages of the complementary switches are not comparable to one another. This is because the MLI makes use of four bidirectional switches and eight unidirectional switches. Accordingly, TSV can be obtained by using Eq. (14).

Total standing voltage (TSV) = 2(V_SA_+ V_SC_) + V_S1_ + V_S3_+ V_S6_ + V_S8_)

= 18V_dc_

14 $$\:{TSV}_{PU}=\frac{{V}_{TSV}}{{V}_{OMAX}}$$

The MBV can be obtained and given below:

MBV_S1_ = MBV_S2_ = MBV_S7_ = MBV_S8_ =3V_dc_.

MBV_S3_ = MBV_S4_ = MBV_S5_ = MBV_S6_ =15V_dc_.

MBV_SA_ = MBV_SB_ =3V_dc_.

MBV_SC_= MBV_SD_=15V_dc_.

The “Normalized voltage stress (NV_strs_)” refers to the ratio of V_strs_ across the IGBT switch to the maximum voltage V_L, max_^31^, given by

15 $$N{V_{strs}} = \frac{{{\bf{Vstrs}}}}{{{\bf{VL}},{\bf{max}}\:}}$$

Where V_strs_ is real voltage stress of the IGBT switch & the values are given in Table 5. The IGBT Switches S_l_, S_2_, S_7_, S_8_, S_A_, and S_B_ experience the lowest V_strs_ and NV_strs_, i.e. 3V_dc_ and 16.66% respectively, whereas switches S_3_, S_4_, S_5_, S_6_, S_C_, and S_D_ experience the highest V_strs_ and NV_strs_, i.e. 15V_dc_ and 83.33%.

**Table 5:** Across power switches, voltage and normalized voltage stress.

Switch	Voltage Stress (V_strs_)	Normalized Voltage Stress (NV_strs_)
S_1_	3V_dc_.	(3V_dc_./18V_dc_) = 16.66%
S_2_	3V_dc_.	(3V_dc_./18V_dc_) = 16.66%
S_3_	15V_dc_.	(15V_dc_./18V_dc_) = 83.33%
S_4_	15V_dc_.	(15V_dc_./18V_dc_) = 83.33%
S_5_	15V_dc_.	(15V_dc_./18V_dc_) = 83.33%
S_6_	15V_dc_.	(15V_dc_./18V_dc_) = 83.33%
S_7_	3V_dc_.	(3V_dc_./18V_dc_) = 16.66%
S_8_	3V_dc_.	(3V_dc_./18V_dc_) = 16.66%
S_A_	3V_dc_.	(3V_dc_./18V_dc_) = 16.66%
S_B_	3V_dc_.	(3V_dc_./18V_dc_) = 16.66%
S_C_	15V_dc_.	(15V_dc_./18V_dc_) = 83.33%
S_D_	15V_dc_.	(15V_dc_./18V_dc_) = 83.33%

**Fig. 9:**
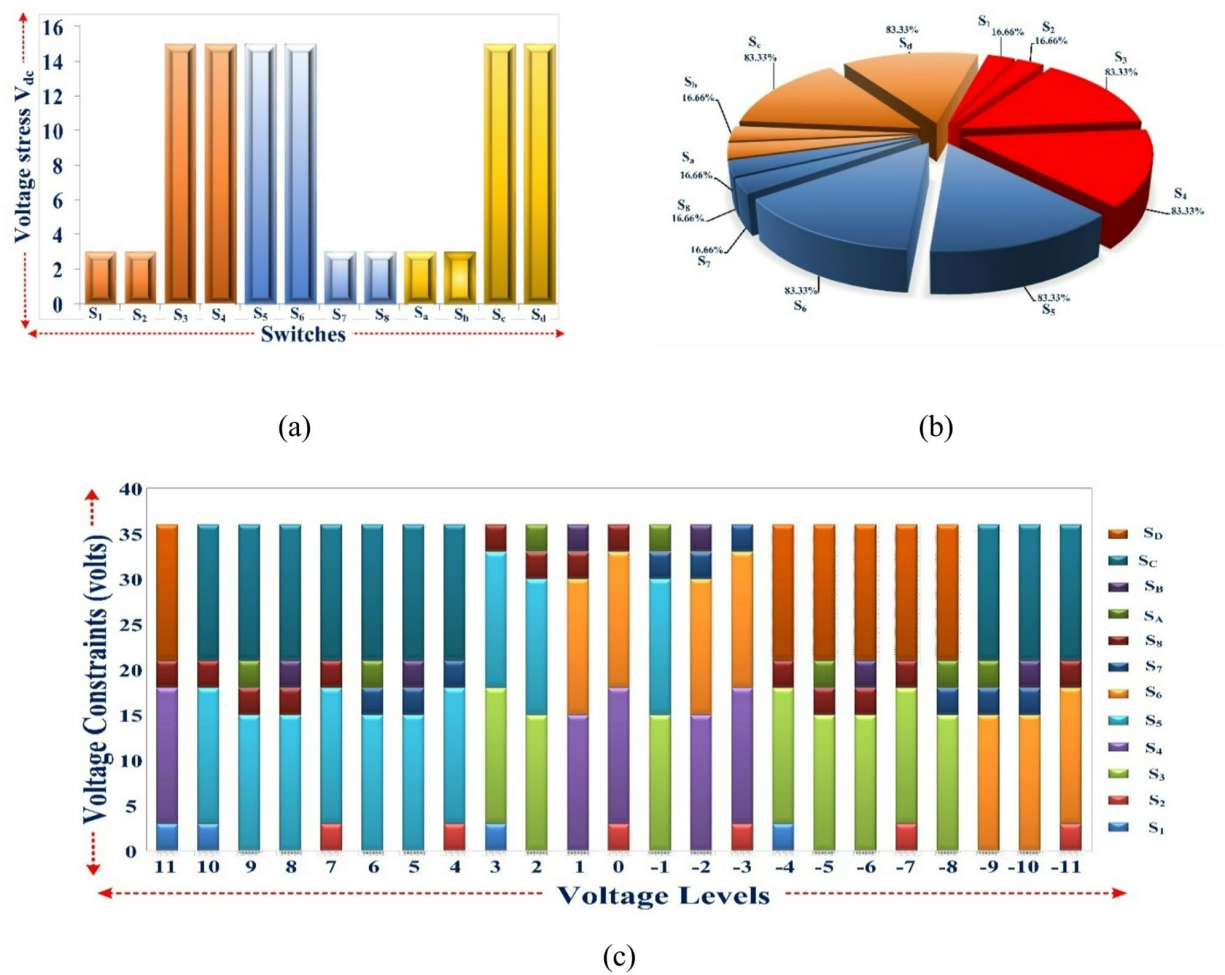
(a) Distribution of voltage stress, (b) % in normalized voltage stress, and (c) Each level’s switches are subject to voltage limitations.

As shown in Fig. 9 (a), Each switch’s stress distribution is illustrated, while Fig. 9 (b) illustrates the normalised voltage stress expressed as a percentage, and Fig. 9 (c) illustrates the voltage limitations that are present in each level of the suggested topology.

### Assessment of the cost function (CF)

The proposed MLI structure’s estimated cost could be affected by a wide range of factors. The sum of all diodes (N_Dio_), switches (N_Swi_), driver circuits (N_Driv_), and sources (N_DS_) is included in this tally. Because of this, the cost component can be figured out by using the following relationship^26^.

16 $$\:CF=\:{(N}_{Swi}+{N}_{Dio}+{N}_{Cap}+\:{N}_{DS}+{N}_{Driv}+\alpha\:{TSV}_{PU})$$

The weight factor ‘α’ is multiplied by the product of ‘TSVpu’. The CF can be calculated by using Eq. (17).

17 $$\:CF=\:{(N}_{Swi}+{N}_{Driv}+{N}_{DS}+\alpha\:{TSV}_{PU})$$

In the majority of instances, the number ought to be greater than 1, even though it ought to be less than 1. To determine the ideal cost factor, the suggested value will be evaluated as 0.5 and 1.5, correspondingly. This will be done to determine the optimal cost factor. According to the computations of the number of components (CF/L), it has been discovered that the MLI is economically possible; the value for CF/level = 0.5 is 1.62, and the value for CF/L = 1.5 is 3.18. Both of these values are based on the MLI. By utilizing Eq. (18), one can determine the component count for each level factor.

18 $$\:{\text{FCC/L = }}\frac{{({N_{Swi}} + {N_{DS}} + {N_{Cap}} + {N_{Dio}} + {N_{Driv}})}}{{Number\:of\:Levels}}$$

### Cost evaluation

To find the larger cost-benefit of using the suggested MLI for lower & medium voltage applications, the maximum operating voltage must be calculated. Given that a switch’s maximum standard commercial voltage is VSW ccv, when using the recommended multilayer inverter, the maximum operating voltage is equivalent to $$\:\raisebox{1ex}{$\sqrt{1.5\:}{\boldsymbol{V}}_{\boldsymbol{S}\boldsymbol{W}\boldsymbol{c}\boldsymbol{c}\boldsymbol{v}}$}\!\left/\:\!\raisebox{-1ex}{$\boldsymbol{\gamma\:}$}\right.$$ where γ is the switch’s safe operating factor, which is typically taken to be 1:7. Therefore, the maximum switch voltage may be used to establish the operation voltage of the proposed structure. When calculating the switch voltage for medium voltage applications, the maximum switch voltage of 3.3 kV is assumed to be the 3-phase operational RMS voltage of 2.3 kV. For 1-φ, the maximum voltage will be 1878 V, while the operational RMS voltage will be 1328 V. Therefore, with an RMS voltage of 1328 V, the voltage magnitudes of the 37-level MLI will be Va = Vb = Vc = 104.33 V, Vd = 730.33 V, and Ve = 834.66 V. Thus, Table V is used to determine the switch rated voltage for the suggested topology, and Table 6 is used to consider the VSW ccv. Table VII calculates and compares the costs of needed IGBTs and driver circuits for 1-φ proposed 37-level MLIs and existing 23-level, 13-level and 11-level MLIs^18,32,33^.

Since device costs fluctuate over time, the values in Table VII are indicative only. For fair comparison across topologies, normalized values such as cost per level (CF/L) are reported in Table VIII, which remain independent of market variations and allow a consistent evaluation of economic feasibility.

**Table 6:** The switches in the suggested topology’s voltage rating.

Switches	Voltage		IGBTs Rating	IGBT model number
	Standard	Normal		
S_1_	313 V	600 V	600 V, 400 A	CM20MD-12 H
S_2_	313 V	600 V	600 V, 400 A	
S_3_	1565 V	1700 V	1700 V, 400 A	CM400DU-34KA
S_4_	1565 V	1700 V	1700 V, 400 A	
S_5_	1565 V	1700 V	1700 V, 400 A	
S_6_	1565 V	1700 V	1700 V, 400 A	
S_7_	313 V	600 V	600 V, 400 A	CM20MD-12 H
S_8_	313 V	600 V	600 V, 400 A	
S_A_	313 V	600 V	600 V, 400 A	
S_B_	313 V	600 V	600 V, 400 A	
S_C_	1565 V	1700 V	1700 V, 400 A	CM400DU-34KA
S_D_	1565 V	1700 V	1700 V, 400 A	

### Comprehensive comparisons

**Fig. 10:**
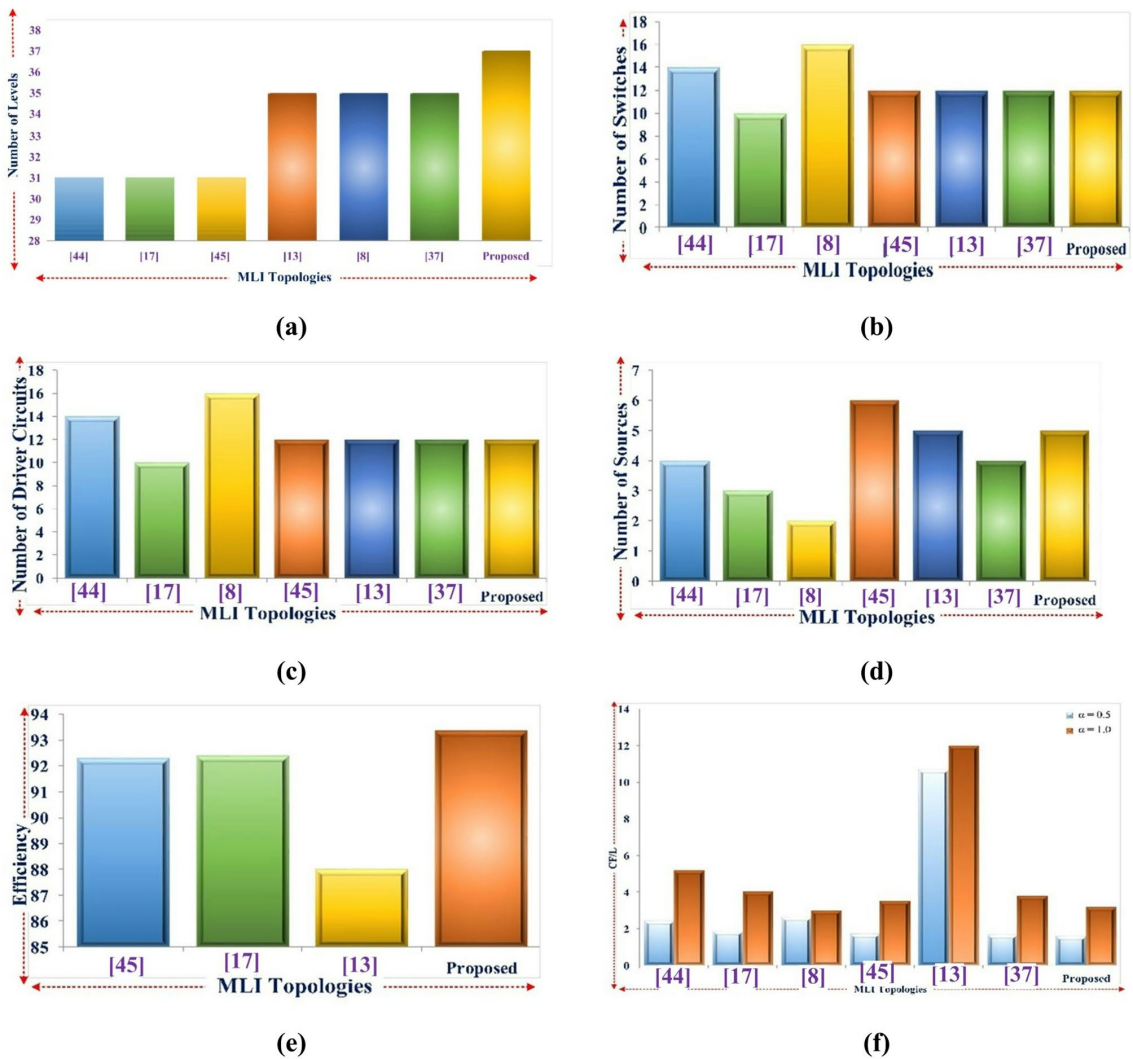
Parameter-based comparisons of the proposed 37- level MLI.

With the assistance of the 37- level MLI that was constructed, Table 7 provides a description and analysis of the topologies that are now in place. Figure 10 (a) examines the number of levels, and Fig. 10 (b) examines the number of DC sources. Both of these comparisons are shown in the figure. A comparison of the total number of driver circuits is shown in Fig. 10(c), a comparison of the number of switches is shown in Fig. 10(d), a comparison of the respective levels of efficiency is shown in Fig. 10(e), and lastly, a comparison of the respective levels of cost function CF/L is shown in Fig. 10(f).

In comparison with the 37-level inverter reported in^45^, the proposed topology achieves the same output levels with reduced device stress, lower TSV, and improved cost-per-level (CF/L). Moreover, hardware validation confirms higher efficiency and lower THD, highlighting the practical advantages of the proposed design over^45^.

**Table 7:** Cost comparison between the proposed topology and existing topologies.

IGBT and Driver model number	Voltage and Current Rating	Unit Cost	Proposed		^34^		^33^		^18^	
			Units	Cost	Units	Cost	Units	Cost	Units	Cost
CM20MD-12 H	600 V, 400 A	$60.77	6	$364.62	-	-	-	-	4	$243.08
CM400HA-24 A	1200 V, 400 A	$118	-	-	1	$118	6	$708	4	$472
CM400DU-34KA	1700 V, 400 A	$516	6	$3096	1	$516	6	$3096	-	-
CM400DY-50 H	2500 V, 400 A	$550	-	-	1	$550	-	-	4	$2200
CM400DY-66 H	3300 V, 400 A	$773	-	-	4	$3092	2	$1546	-	-
844-SD303C25S20C	2500 V, 350 A	$102.5	-	-	3	$307.50	-	-	-	-
SKYPER-32PRO2	Up to 1700 V	$92.71	12	$1112.52	1	$92.71	6	$556.26	8	$741.68
1SC0450V2A0-65	Up to 6500 V	$267.6	-	-	3	$802.86	1	$267.62	4	$1070.48
Overall cost			$4573.14		$5479.07		$6173.88		$4727.24	
Number of voltage output levels			37		11		13		23	

Courtesy: www.nevonexpress.com, www.yaspro.com, * Prices may vary.

**Table 8:** Parameter-based comparisons of the proposed MLI.

Topologies	*N*_SWI_	*N*_DCS_	*N*_LEV_	*N*_CAP_	*N*_DRK_	TSV (v)	Efficiency	CF/L	
								α = 1.5	α = 0.5
^44^	14	4	31	-	14	86	92.34	5.24	2.46
^17^	10	3	31	2	10	67	92.6	4.03	1.87
^45^	12	2	31	4	12	84	NR	3.04	2.64
^13^	12	6	35	-	12	63	NR	3.52	1.72
^8^	16	5	35	2	16	92	87.8	11.8	10.2
^37^	12	4	35	-	12	52	NR	3.19	1.68
^46^	14	5	37	-	14	92	91.8	4.85	2.31
Proposed	12	5	37	-	12	55	93.26	3.18	1.62

*NR = Metrics are not reported in the cited source.

## Conclusion

In this study, an asymmetric 37-level MLI was proposed with the goal of lowering the price of MLIs, as well as their size and overall harmonic distortion. The MLI that has been recommended not only reduces the costs of maintenance but also achieves higher levels of efficiency and dependability. The 37- level inverter architecture that was presented is supported by the creation of the simulation model in MATLAB/Simulink as well as the validation using experimental outcomes. A comparison is made between the features and a wide variety of other complex topologies after the analysis has been completed. In comparison, the TSV voltage of MLI is 18V_DC_, the efficiency is 93.26%, the CF/L is 1.62 and 3.18 for = 0.5 and = 1.5, respectively, and the THD is 1.21%. The proposed inverter’s cost is far lower than it was in the more recent topologies. Multiple applications may be found for the proposed MLI structure that include active filters, Dynamic voltage restorers (DVRs), Electric Vehicles (EVs), Uninterruptible Power Supplies (UPS), and grid-connected Renewable Energy Systems (RES).

## Data Availability

The data used to support the findings of this study are included in the article.

## References

[1] Mondol, M. et al. A new integrated multilevel inverter topology for renewable energy transformation. *IEEE Trans. Ind. Appl.* **59** (3), 3031–3043 (2023).

[2] Poorfakhraei, A., Narimani, M. & Emadi, A. A review of multilevel inverter topologies in electric vehicles: current status and future trends. *IEEE Open. J. Power Electron.* **2**, 155–170 (2021).

[3] Narasipuram, R. et al. The electric vehicle surge: effective solutions for charging challenges with advanced converter technologies. *Energy Eng.* **122**, 2 (2025).

[4] Suthar, A. N., Venkataramanaiah, J. & Suresh, Y. Conventional, wide-bandgap, and hybrid power converters: A comprehensive review. *Renew. Sustain. Energy Rev.* **213**, 115419 (2025).

[5] Mukundan, N. et al. A new multilevel inverter based grid connected reliable solar power transfer unit with power quality enhancement. *IEEE Trans. Ind. Appl.* **59** (2), 1887–1900 (2022).

[6] Wang, D. et al. Multilevel inverters for electric aircraft applications: current status and future trends. *IEEE Trans. Transp. Electrification*. **10** (2), 3258–3282 (2023).

[7] Sarebanzadeh, M. et al. A 15-level switched-capacitor multilevel inverter structure with self-balancing capacitor. *IEEE Trans. Circuits Syst. II Express Briefs*. **69** (3), 1477–1481 (2021).

[8] Tapas Roy, P. K., Sadhu, A. & Dasgupta Cross-switched multilevel inverter using novel switched capacitor converters. *IEEE Trans. Ind. Electron.* **66** (11), 8521–8532 (2019).

[9] Munawar, S. et al. Multilevel inverters design, topologies, and applications: research issues, current, and future directions. *IEEE Access.* (2024).

[10] Sharma, B. et al. A comprehensive review of multi-level inverters, modulation, and control for grid-interfaced solar PV systems. *Sci. Rep.* **15** (1), 661 (2025). 39753791 10.1038/s41598-024-84296-1 PMC11698899

[11] Mansourizadeh, H. et al. A 13-level switched-capacitor-based multilevel inverter with reduced components and inrush current limitation. *Sci. Rep.* **15** (1), 290 (2025). 39747294 10.1038/s41598-024-84148-y PMC11697303

[12] Jayaraman, R. et al. High-efficiency multilevel inverter topology with minimal switching devices for enhanced power quality and reduced losses. *IET Power Electron.* **18** (1), e12851 (2025).

[13] Fatemeh & Esmaeili Kazem Varesi, An Asymmetric Multi-Level Inverter Structure with Increased Steps per Devices, in: In 2020 11th Power Electronics, Drive Systems, and Technologies Conference (PEDSTC), pp. 1–5. IEEE. (2020).

[14] Elias, M. F. & Mohamad Nasrudin Abd Rahim, and Nur Farhana Rosli. A three-phase hybrid multilevel inverter with enhanced pulse-width modulation strategy. *IEEE Trans. Power Electron.* **38** (4), 4714–4726 (2022).

[15] Venkataramanaiah, J. & Suresh, Y. Design and development of a novel 19-level inverter using an effective fundamental switching strategy. *IEEE J. Emerg. Sel. Top. Power Electron.* **6**(4), 1903–1911 (2017).

[16] Roy, T. & Sadhu, P. K. A step-up multilevel inverter topology using novel switched capacitor converters with reduced components. *IEEE Trans. Ind. Electron* **68**(1), 236–247 (2021).

[17] Hussan, M. R. et al. A novel switched-capacitor multilevel inverter topology for energy storage and smart grid applications. *Electron. (Basel)*. **9** (10), 1703 (2020).

[18] Karania, N. et al. Hybrid control system for cascaded H-bridge multi-level inverter based shunt active filter for photovoltaic generation. *Electr. Power Syst. Res.* **241**, 111248 (2025).

[19] Venkataramanaiah, J. et al. A new method for selecting optimum levels in asymmetric Cascaded H-Bridge‐Multilevel Inveter with variable DC sources. *Int. J. Circuit Theory Appl.* **53**(2), 1056–1071 (2025).

[20] Jayakumar, V. & Chokkalingam, B. Lange Munda. A comprehensive review on space vector modulation techniques for neutral point clamped multi-level inverters. *IEEE Access.* **9**, 112104–112144 (2021).

[21] Monteiro, A. et al. Cascaded transformers-based multilevel inverters with Npc. *IEEE Trans. Industr. Electron.* **69**, 7879–7889 (2021).

[22] Zakzewski, D., Resalayyan, R. & Khaligh, A. Hybrid neutral point clamped converter: review and comparison to traditional topologies. *IEEE Trans. Transp. Electrification*. **10** (3), 6087–6099 (2023).

[23] Sadanala, C., Pattnaik, S. & Vinay Pratap Singh A flying capacitor-based multilevel inverter architecture with symmetrical and asymmetrical configurations. *IEEE J. Emerg. Sel. Top. Power Electron.* **10** (2), 2210–2222 (2020).

[24] Khoshkbar-Sadigh, A., Dargahi, V. & Corzine, K. New flying-capacitor-based multilevel converter with optimized number of switches and capacitors for renewable energy integration. *IEEE Trans. Energy Convers.* **31** (3), 846–859 (2016).

[25] Ding, J. et al. A novel multilevel converter as active magnetic bearing drive based on hybrid NPC with Flying-Capacitor leg. *IEEE Trans. Industr. Electron.* (2025).

[26] Goel, R., Davis, T. T. & Dey, A. Thirteen-level multilevel inverter structure having single DC source and reduced device count. *IEEE Trans. Ind. Appl.* **58** (4), 4932–4942 (2022).

[27] Sridhar, V. & Umashankar, S. A comprehensive review on CHB MLI based PV inverter and feasibility study of CHB MLI based PV-STATCOM. *Renew. Sustain. Energy Rev.* **78**, 138–156 (2017).

[28] Singh, A. et al. Enhancing power quality in electric vehicles and battery energy storage systems using multilevel inverter topologies–A review. *J. Energy Storage*. **110**, 115274 (2025).

[29] Tuhin, N. S. Design and Performance Analysis of a Novel Asymmetrical Multilevel Inverter Structure With Reduced Components Using the Half-Height Modulation Technique. *International Transactions on Electrical Energy Systems* **2025**(1), 5546944 (2025).

[30] Ardashir, J. et al. *Integration of Energy Storage Systems with Multilevel Inverters for microgrids. Distributed Energy Storage Systems for Digital Power Systems* 293–318 (Elsevier, 2025).

[31] Lin, C. H. et al. Performance enhancement of a multilevel inverter in renewable energy systems using equilibrium optimizer. *Electr. Power Syst. Res.* **243**, 111538 (2025).

[32] Mohammad, K. et al. Proposed high gain single DC-source SC-MLI topology for solar PV grid integration applications. *Int. J. Power Electron. Drive Syst. (IJPEDS)* 16 .1 : 344–354. (2025).

[33] Tiwari, A. & Agarwal, R. Single DC-link-based 5-level MLI topology for renewable and grid applications with fewer switches. *Electr. Eng.* **106** (4), 3883–3898 (2024).

[34] Tejasvi, B. & Vijayapriya, P. A comprehensive review of various MLI topologies to minimise the THDs for FACTS applications. *Int. J. Syst. Syst. Eng.* **14** (4), 403–445 (2024).

[35] Farhangi, M. et al. A single-source single-stage switched-boost multilevel inverter: Operation, topological extensions, and experimental validation. *IEEE Trans. Power Electron.* **37** (9), 11258–11271 (2022).

[36] Kazem Varesi, M., Karimi, P. & Kargar A new basic step-up cascaded 35-level topology extendable to higher number of levels, in: In 2019 10th International Power Electronics, Drive Systems and Technologies Conference (PEDSTC), pp. 291–296. IEEE. (2019).

[37] Dhanamjayulu, C., Sanjeevikumar, P. & Muyeen, S. M. A structural overview on transformer and transformer-less multi level inverters for renewable energy applications. *Energy Rep.* **8**, 10299–10333 (2022).

[38] Harbi, I. et al. Model-predictive control of multilevel inverters: Challenges, recent advances, and trends. *IEEE Trans. Power Electron.* **38** (9), 10845–10868 (2023).

[39] Guo, X. et al. Improved modulation strategy for singe-phase cascaded H-bridge multilevel inverter. *IEEE Trans. Power Electron.* **37** (3), 2470–2474 (2021).

[40] Júnior, S. C. et al. Asymmetric 49-levels cascaded MPUC multilevel inverter fed by a single DC source. *IEEE Trans. Ind. Appl.* **58** (6), 7539–7549 (2022).

[41] Roy, T. et al. A 7-level switched capacitor multilevel inverter with reduced switches and voltage stresses. *IEEE Trans. Circuits Syst. II Express Briefs*. **68** (12), 3587–3591 (2021).

[42] Kishore, N., Shukla, K. & Gupta, N. A novel three-phase 13-level cascaded hybrid-module based multilevel inverter with level-shifted modified-PWM algorithm. *IEEE Trans. Ind. Appl.* **60** (2), 3263–3272 (2023).

[43] Devalraju Prasad, C., Dhanamjayulu, S., Padmanaban, J. B. & HolmNielsen Frede Blaabjerg, Shaik Reddi Khasim, design and implementation of 31- level asymmetrical inverter with reduced components. *IEEE Access.* **9**, 22788–22803 (2021).

[44] Kazem Varesi, M., Karimi, P. & Kargar, A. N. Cascaded 35-Level Inverter with Reduced Switch Count, in: In 2019 Iranian Conference on Renewable Energy & Distributed Generation (ICREDG), pp. 1–5. IEEE. (2019).

[45] Dhanamjayulu, C. Design of 37-Level inverter with reduced switch count for low total harmonic Distortion. In 2023 Innovations in Power and Advanced Computing Technologies (i-PACT), 1–8. IEEE, (2023).

[46] Debela, T., Singh, J. & Vijay, K. Sood. Evaluation of a grid-connected reduced‐component boost multilevel inverter (BMLI) topology. *Int. J. Circuit Theory Appl.* **50** (6), 2075–2107 (2022).

[47] Debela, T., Singh, J. & Vijay, K. Sood. An assessment of H-bridge less grid‐tied multilevel inverter with minimum device count and lesser total standing voltage. *IET Power Electronics* (2023).

